# Heterogeneity in health status and the influence of patient characteristics across patients seeking musculoskeletal orthopaedic care – a cross-sectional study

**DOI:** 10.1186/1471-2474-14-83

**Published:** 2013-03-07

**Authors:** Anthony V Perruccio, Rajiv Gandhi, Y Raja Rampersaud

**Affiliations:** 1 Division of Orthopaedic Surgery, Toronto Western Hospital, University Health Network, 1st Floor, East Wing, Room 441, Toronto, ON, Canada; 2 Institute of Health Policy, Management and Evaluation, University of Toronto, Toronto, ON, Canada; 3 Toronto Musculoskeletal Centre, University of Toronto, Toronto, ON, Canada; 4 Department of Surgery, Faculty of Medicine, University of Toronto, Toronto, ON, Canada

**Keywords:** Health status, Patient characteristics, Orthopaedics, Patient education

## Abstract

**Background:**

Health status is an important predictor of patient outcomes. Consequently, identifying patient predictors of health status is essential. In musculoskeletal orthopaedic care, the majority of work examining the association between patient characteristics and health status has been undertaken among hip/knee cohorts. We investigate these associations comparing findings across four musculoskeletal cohorts (hip/knee; foot/ankle; neck/back; elbow/shoulder).

**Methods:**

Patients seeking elective musculoskeletal orthopaedic care were recruited prior to consultation. Questionnaires captured health domain status (bodily pain, physical functioning, and mental and general health) and covariates: demographics; socioeconomic characteristics; and comorbidity. Scores were compared across cohorts. Two path regression analyses were undertaken. First, domain scores were simultaneously examined as dependent variables in the overall sample. Subsequently, the model was assessed stratified by cohort.

**Results:**

1,948 patients: 454 neck/back, 767 hip/knee, 378 shoulder/elbow, 349 foot/ankle. From stratified analyses, significant variability in covariate effects was observed. Worse bodily pain scores were associated with increasing age and female sex among hip/knee, low income among foot/ankle, and overweight/obese for foot/ankle and hip/knee. Worse mental health scores were associated with low income across cohorts except elbow/shoulder, low education within neck/back, and compared to Whites, Blacks had significantly worse scores among foot/ankle, better scores among hip/knee. Worse general health scores were observed for Asians among hip/knee, Blacks among foot/ankle, and South-Asians among elbow/shoulder and neck/back.

**Conclusion:**

The substantial heterogeneity across musculoskeletal cohorts suggests that patient- and cohort-specific approaches to patient counsel and care may be more effective for achieving optimal health and outcomes.

## Background

Elective orthopaedic surgical care for musculoskeletal disorders comprises strategies to optimize patient health and related quality of life, patient education, and shared decision-making evaluating the risks and benefits to surgical and non-surgical treatment options [1]. Success in this endeavour hinges on a great extent to understanding the patient population and critically recognizing how patient characteristics relate to and may influence health status. Generally, the literature has identified variability in the association between individual patient characteristics and patient-reported health status, willingness to consider surgery, and satisfaction with care [2-14]. To date, there has been limited work in this area within musculoskeletal orthopaedic care, and the focus has primarily been on hip and knee replacement cohorts. Whether associations between personal patient characteristics and patient-reported health status are similar for non-hip and -knee cohorts is unknown, however. This knowledge gap potentially minimizes the effectiveness of strategies related to patient education, efforts to modify health behaviours, and shared decision-making should differences exist. Therefore, a developed understanding of these relationships is essential to enable clinicians to truly tailor strategies such that individual patients ultimately realize maximal health outcomes and satisfaction with their decisions and care.

To our knowledge, there has not been a study which has directly compared patient-reported health status across patients seeking musculoskeletal orthopaedic care for different anatomical regions. Further, no investigations have assessed whether the associations between personal characteristics and health status vary across these patient cohorts. While comparisons of health outcomes across different studies can be helpful, comparability is enhanced when the groups are examined within the context of the same study with identical measurement of health status and analytic strategy. In this study, we focused on comparing patient-reported health status measures across four cohorts seeking musculoskeletal orthopaedic care (foot/ankle; elbow/shoulder; neck/back; hip/knee). We investigated whether differences in health statuses were explained by differential distributions of personal patient characteristic between the cohorts, and determined whether the associations between these characteristics and health measures varied across cohorts.

## Methods

This cross-sectional study consecutively recruited individuals seeking elective musculoskeletal orthopaedic care while waiting for a consultation at the orthopaedic ambulatory clinic of an academic hospital in Toronto Canada from 2008 to 2010. Eligibility criteria included ages ≥18 years, diagnosis of degenerative hip or knee, shoulder or elbow, or foot or ankle pathology and degenerative neck or back disc pathology or spinal stenosis (including degenerative spondylolisthesis). As well, individuals had to have sufficient fluency in English to complete the questionnaire, not be institutionalized in non-voluntary and/or dependent residence, competent to give informed consent, and not suffering from any emergent-musculoskeletal, traumatic, myelopathy-related, or inflammatory conditions to be eligible. The study was approved by the University Health Network Research Ethics Board. Written informed consent was obtained from all study participants.

Consenting patients completed a questionnaire prior to consultation. All responses were self-reported. Participating surgeons confirmed, post-consultation, whether eligibility/exclusion criteria were met. The questionnaire included the Medical Outcomes Short Form-36 (SF-36) [15], and also captured patient demographics, socioeconomic characteristics, and comorbid conditions.

The SF-36 was specifically chosen as it is the most widely used generic health measure, not targeted to specific ages, diseases, or treatment groups. It has documented reliability and validity in general population and clinical samples, and across varied conditions [15-17]. We focused on four health domains measured by the SF-36. *Physical functioning*, comprised of 10 items, assessed the extent to which an individuals’ health limited vigorous or moderate activities such as running, lifting, moving, climbing, and walking. *Bodily pain*, a 2-item scale, assessed the amount of pain and the extent to which the pain interfered with normal work activities (both in and outside the home). *General health,* a 5-item scale, assessed patient perceptions of their overall health status. Finally, *Mental health*, a 5-item scale, assessed psychological distress and well-being. Each of the health domain scores was standardized to a 0–100 scale, with lower scores representing worse health/well-being.

The questionnaire also captured patient *age*, *sex*, *household income* (low (<$45,000); middle (45,000 - <60,000); high (≥$60,000); and as nearly 18% of the sample did not provide income, a missing category also was retained), *level of education* (≤high school graduation; post-secondary), *racial background* (White; Asian; Black; South Asian; Other), height and weight, used to calculate body mass index (BMI) (kg/m^2^; normal (BMI ≤ 24.99); overweight (25 < =BMI < =29.99); obese (BMI ≥ 30), and number of comorbid chronic conditions (based on self-reported indications from a list of 18 conditions, with the option of reporting others).

### Statistical Methods

Sample descriptives were examined for the overall sample and by cohort. Statistical comparisons across cohorts were made by way of analysis of variance, chi-square test, or Kruskal-Wallis Test as appropriate.

Two sets of path regression analyses were undertaken. In the first set, all health domain scores were simultaneously examined as dependent variables in the overall sample. An indicator variable identifying the cohort was specified as a predictor variable (reference: hip/knee), along with age, sex (reference: male), level of income (reference: high) and education (reference: ≥post-secondary), race/ethnicity (reference: White), comorbidity count, and overweight and obesity (reference: normal BMI). Cognizant that the health status outcomes are interrelated to some degree (beyond the effects of the predictor variables), the outcomes were specified to co-vary in the model.

To identify any differences across cohorts, the second set of regressions investigated the same model but with the analyses stratified by cohort. Again, health domain scores were specified to co-vary.

Analyses were carried out using Mplus 6.1 [18] using full information maximum likelihood. Conservatively, sample size determination was based on the stratified model, with the requirement of at least five subjects per parameter to be estimated in covariance structure modeling [19,20]. Based on our stratified model, comprised of 65 parameters, this required an n ≥ 325 within each cohort. For the smallest surgical cohort (foot/ankle (n = 349)), the available n provided 20.5 cases per variable in the model.

## Results

Two-thousand patients completed the survey with valid data from 1,948 patients: 454 (23%) for neck/back, 767 (39%) for hip/knee, 378 (20%) for shoulder/elbow, and 349 (18%) for foot/ankle. Significant differences were observed in sex, education, and overweight/obese proportions across the cohorts (Table 1). The greatest proportion of women was found among the foot/ankle group, low educational attainment among the neck/back cohort, and greatest proportion of overweight/obese among the hip/knee cohort. Income differences were marginally non-significant across cohorts, with the proportion of low household income greatest among the shoulder/elbow cohort. Significant age differences were observed across cohorts, with the highest mean age (55.5 years) among the hip/knee. The difference between the oldest (hip/knee) and youngest cohorts (foot/ankle and neck/back) was 6 years, and age ranges were similar across the groups.

**Table 1:** Sample demographic and health characteristics, overall and by surgical cohort, including tests of differences across cohorts

		**Overall sample (n = 1948)**	**Foot/Ankle (n = 349)**	**Elbow/Shoulder (n = 378)**	**Neck/Back (n = 454)**	**Hip/Knee (n = 767)**	**p-value**
**Proportions (%)**							
Sex	Female	48.6	60.7	39.2	48.1	48.1	**<0.0001**
Income	Low	23.2	22.4	27.1	19.4	23.8	**<0.0001**
	Missing	17.7	11.9	11.5	30.6	15.8	
Education	<= high school	32.6	21.6	33.4	37.8	34.1	**<0.0001**
Race	White	78.4	81.1	74.8	79.2	78.3	0.1897
	Asian	5.0	4.2	5.3	5.6	4.9	
	Black	4.5	2.8	5.3	4.1	5.0	
	South Asian	4.3	3.9	4.0	4.1	4.9	
	Other	7.8	8.1	10.6	6.9	6.9	
Body Mass Index	Overweight	38.0	34.3	40.6	36.5	39.5	**<0.0001**
	Obese	24.7	19.8	23.4	21.5	29.5	
**Means (sd)**							
Age		51.9 (18–93)	49.0 (18–84)	50.7 (18–90)	49.0 (18–87)	55.5 (18–93)	**<0.0001**
Comorbidity Count		1.5 (1.9)	1.5 (1.9)	1.3 (1.7)	1.5 (1.9)	1.7 (1.9)	**0.0011**
Body Mass Index		27.2 (5.4)	26.3 (5.3)	27.0 (4.7)	26.8 (5.5)	27.9 (5.6)	**<0.0001**
Bodily Pain		40.5 (23.5)	47.9 (24.3)	42.5 (23.7)	37.0 (23.7)	38.1 (22.1)	**<0.0001**
Physical Functioning		52.2 (28.6)	60.7 (28.6)	66.1 (25.2)	47.3 (27.8)	44.4 (27.1)	**<0.0001**
Mental Health		68.7 (20.3)	71.4 (18.9)	70.2 (19.8)	65.0 (21.1)	69.0 (20.3)	**<0.0001**
General Health		66.3 (21.1)	68.9 (21.3)	67.9 (20.3)	61.1 (22.0)	67.3 (20.4)	**<0.0001**

Significant differences across the cohorts were found across all health domain scores (Table 1). Lower mean scores (i.e. worse scores) were found among the neck/back group for bodily pain, among the hips/knees for physical functioning, and among the neck/back for mental and general health.

From combined analyses (Table 2), adjusted for demographic factors, comorbidity and BMI, neck/back patients had worse health domain scores, except for physical functioning, compared to hip/knee patients. Compared to the hip/knee group, foot/ankle patients had better health domain scores, except for general health. The elbow/shoulder group had similar health domain scores to the hip/knee group, except for physical functioning where better scores were observed in the elbow/shoulder group.

**Table 2:** Path regression results; cohorts considered together*

		Dependent variables: health domain scores*			
		**Bodily pain**	**Physical func.**	**Mental health**	**General health**
Predictors			Beta Coefficients**		
			(95% Confidence Limits)		
Age		**−0.14**	**−0.31**	**0.20**	0.04
		(−0.21, -0.07)	(−0.39, -0.23)	(0.14, 0.26)	(−0.02, 0.10)
Sex (ref: Male)	Female	**−2.99**	**−3.68**	−1.37	0.37
		(−5.14, -0.85)	(−6.08, -1.27)	(−3.21, 0.46)	(−1.50, 2.23)
Income (ref: high)	Low	**−6.71**	**−7.54**	**−7.87**	**−5.52**
		(−9.97, -3.46)	(−11.18, -3.90)	(−10.66, -5.07)	(−8.35, -2.69)
	Missing	1.51	−0.19	−1.67	**−2.61**
		(−1.35, 4.36)	(−3.38, 3.01)	(−4.11, 0.77)	(−5.08, -0.14)
Education (ref: >high school)	<= high school	**−4.36**	**−5.18**	**−3.36**	**−2.55**
		(−6.65, -2.07)	(−7.75, -2.61)	(−5.32, -1.40)	(−4.54, -0.57)
Race (ref: White)	Asian	4.47	1.49	3.12	**−5.10**
		(−0.32, 9.25)	(−3.88, 6.86)	(−0.97, 7.21)	(−9.26, -0.95)
	Black	−2.35	−2.53	−1.12	**−5.48**
		(−7.53, 2.84)	(−8.34, 3.28)	(−5.60, 3.35)	(−10.01, -0.95)
	Other	0.92	0.48	−0.62	−1.39
		(−3.01, 4.84)	(−3.92, 4.88)	(−3.99, 2.75)	(−4.81, 2.02)
	South Asian	−0.88	**−7.50**	−0.55	**−4.69**
		(−5.96, 4.19)	(−13.15, -1.86)	(−4.88, 3.77)	(−9.07, -0.32)
Comorbidity Count		**−2.11**	**−2.36**	**−2.83**	**−3.87**
		(−2.70, -1.52)	(−3.01, -1.70)	(−3.33, -2.32)	(−4.38, -3.36)
Body Mass Index (ref: Normal)	Overweight	**−3.49**	**−3.53**	0.34	−0.89
		(−5.93, -1.05)	(−6.27, -0.80)	(−1.75, 2.42)	(−3.01, 1.22)
	Obese	**−7.28**	**−8.84**	0.28	**−5.44**
		(−10.03, -4.54)	(−11.92, -5.75)	(−2.08, 2.63)	(−7.83, -3.06)
Cohort (ref: Hip/Knee)	Foot/Ankle	**7.18**	**12.27**	**2.86**	0.28
		(4.22, 10.14)	(8.94, 15.59)	(0.32, 5.40)	(−2.29, 2.85)
	Elbow/Shoulder	1.95	**18.38**	0.48	−0.87
		(−0.96, 4.85)	(15.12, 21.65)	(−2.00, 2.97)	(−3.38, 1.65)
	Neck/Spine	**−3.40**	−0.39	**−2.87**	**−6.34**
		(−6.15, -0.64)	(−3.49, 2.70)	(−5.23, -0.52)	(−8.74, -3.95)

*all health domain scores were simultaneously assessed as dependent variables, with allowance for correlations between health domain scores;

**bolded estimates represent statistical significance (i.e. p < 0.05).

Increasing age was associated with worse bodily pain and physical functioning scores, and better mental health scores. Women had worse scores for bodily pain and physical functioning but no sex differences were observed for other health domains. Consistently, a lower level of household income and educational attainment was associated with worse health domain scores. Compared to Whites, Blacks had significantly worse general health scores, and South Asians worse physical functioning and general health scores. Across cohorts, increasing comorbidity was associated with worse health domain scores. Finally, obesity was associated with worse scores for all health domains, except mental health.

From stratified analyses, a number of differential effects on the health domain scores were noted across the cohorts. Results are presented in Tables 3, 4, 5, 6 for each of the four health domains, respectively.

**Table 3:** Cohort-stratified path regression results; outcome: bodily pain score*

		**Foot/Ankle**	**Elbow/Shoulder**	**Neck/Back**	**Hip/Knee**
		**(n = 349)**	**(n = 378)**	**(n = 454)**	**(n = 767)**
			Outcome: Bodily Pain Score		
Predictors			Beta Coefficients**		
			(95% Confidence Limits)		
					
Age		−0.14	−0.04	−0.13	**−0.23**
		(−0.31, 0.03)	(−0.21, 0.13)	(−0.28, 0.03)	(−0.34, -0.12)
Sex (ref: Male)	Female	−0.25	−4.07	−1.33	**−5.06**
		(−5.46, 4.96)	(−9.18, 1.04)	(−6.03, 3.37)	(−8.27, -1.86)
Income (ref: high)	Low	**−11.42**	−4.95	−7.55	−4.41
		(−18.97, -3.88)	(−12.38, 2.49)	(−15.26, 0.16)	(−9.33, 0.52)
	Missing	2.92	5.05	2.85	−1.56
		(−5.15, 11.00)	(−3.03, 13.13)	(−2.09, 7.78)	(−6.07, 2.96)
Education (ref: >high school)	≤ high school	−5.58	−4.46	−3.88	**−3.58**
		(−11.74, 0.58)	(−9.70, 0.78)	(−8.61, 0.85)	(−7.10, -0.06)
Race (ref: White)	Asian	1.91	**16.07**	3.33	2.34
		(−10.65, 14.47)	(4.85, 27.28)	(−6.85, 13.51)	(−4.72, 9.40)
	Black	−3.84	−0.87	−5.84	−0.36
		(−19.78, 12.09)	(−13.33, 11.60)	(−17.46, 5.78)	(−7.33, 6.61)
	Other	1.77	2.34	−2.62	2.01
		(−7.37, 10.91)	(−5.71, 10.38)	(−11.69, 6.45)	(−4.21, 8.24)
	South Asian	−1.36	0.68	2.61	−2.81
		(−13.95, 11.22)	(−12.34, 13.71)	(−8.49, 13.71)	(−10.00, 4.38)
Comorbidity Count		**−1.64**	**−3.47**	**−1.95**	**−1.71**
		(−3.09, -0.19)	(−5.02, -1.92)	(−3.18, -0.72)	(−2.56, -0.85)
Body Mass Index (ref: Normal)	Overweight	**−6.47**	3.23	−3.60	**−4.99**
		(−12.25, -0.69)	(−2.43, 8.89)	(−8.79, 1.60)	(−8.76, -1.22)
	Obese	**−13.27**	−6.19	−3.54	**−7.12**
		(−20.00, -6.54)	(−12.82, 0.43)	(−9.69, 2.60)	(−11.15, -3.09)

*all health domain scores were simultaneously assessed as dependent variables, with allowance for correlations between health domain scores;

**bolded estimates represent statistical significance (i.e. p < 0.05).

**Table 4:** Cohort-stratified path regression results; outcome: physical functioning score*

		**Foot/Ankle**	**Elbow/Shoulder**	**Neck/Back**	**Hip/Knee**
		**(n = 349)**	**(n = 378)**	**(n = 454)**	**(n = 767)**
			Outcome: Physical Functioning Score		
Predictors			Beta Coefficients**		
			(95% Confidence Limits)		
Age		**−0.25**	**−0.20**	**−0.34**	**−0.38**
		(−0.45, -0.04)	(−0.37, -0.03)	(−0.51, -0.17)	(−0.51, -0.25)
Sex (ref: Male)	Female	5.13	**−6.85**	−4.06	**−6.20**
		(−0.96, 11.22)	(−11.88, -1.82)	(−9.28, 1.17)	(−9.93, -2.46)
Income (ref: high)	Low	**−14.29**	**−10.28**	−0.56	−5.42
		(−23.07, -5.50)	(−17.61, -2.96)	(−9.08, 7.96)	(−11.15, 0.30)
	Missing	−1.56	−6.34	2.86	0.21
		(−10.99, 7.87)	(−14.19, 1.51)	(−2.65, 8.37)	(−5.05, 5.47)
Education (ref: >high school)	≤ high school	−6.59	−5.12	**−5.50**	**−4.28**
		(−13.78, 0.60)	(−10.28, 0.04)	(−10.77, -0.22)	(−8.39, -0.18)
Race (ref: White)	Asian	−3.13	4.47	4.10	0.92
		(−17.80, 11.54)	(−6.59, 15.53)	(−7.16, 15.35)	(−7.31, 9.16)
	Black	3.80	−10.12	−2.21	−0.18
		(−14.83, 22.42)	(−22.12, 1.88)	(−15.21, 10.79)	(−8.31, 7.96)
	Other	5.65	1.17	−5.05	−0.13
		(−5.04, 16.33)	(−6.82, 9.16)	(−15.19, 5.09)	(−7.30, 7.04)
	South Asian	−6.46	−6.70	−12.17	−4.17
		(−21.17, 8.26)	(−19.29, 5.89)	(−24.59, 0.25)	(−12.42, 4.08)
Comorbidity Count		−1.10	**−3.13**	**−1.79**	**−2.61**
		(−2.79, 0.59)	(−4.66, -1.60)	(−3.15, -0.42)	(−3.60, -1.62)
Body Mass Index (ref: Normal)	Overweight	−5.54	2.79	**−6.39**	−2.90
		(−12.30, 1.22)	(−2.78, 8.37)	(−12.19, -0.59)	(−7.29, 1.49)
	Obese	**−14.31**	−5.09	**−9.36**	**−7.24**
		(−22.18, -6.45)	(−11.63, 1.45)	(−16.22, -2.50)	(−11.92, -2.55)

*all health domain scores were simultaneously assessed as dependent variables, with allowance for correlations between health domain scores;

**bolded estimates represent statistical significance (i.e. p < 0.05).

**Table 5:** Cohort-stratified path regression results; outcome: mental health score*

		**Foot/Ankle**	**Elbow/Shoulder**	**Neck/Back**	**Hip/Knee**
		**(n = 349)**	**(n = 378)**	**(n = 454)**	**(n = 767)**
			Outcome: Mental Health Score		
Predictors			Beta Coefficients**		
			(95% Confidence Limits)		
Age		**0.19**	**0.20**	**0.30**	**0.13**
		(0.06, 0.31)	(0.06, 0.33)	(0.17, 0.43)	(0.03, 0.23)
Sex (ref: Male)	Female	1.03	−3.54	−2.70	−1.00
		(−2.83, 4.89)	(−7.66, 0.57)	(−6.76, 1.35)	(−3.97, 1.98)
Income (ref: high)	Low	**−9.37**	**−8.82**	−4.51	**−7.40**
		(−14.96, -3.79)	(−14.80, -2.84)	(−11.18, 2.16)	(−12.00, -2.80)
	Missing	−2.00	−5.34	0.04	−1.87
		(−7.95, 3.95)	(−11.88, 1.20)	(−4.21, 4.30)	(−6.08, 2.34)
Education (ref: >high school)	≤ high school	−3.11	−2.74	**−4.19**	−2.79
		(−7.69, 1.47)	(−6.96, 1.48)	(−8.27, -0.10)	(−6.07, 0.49)
Race (ref: White)	Asian	4.39	3.61	6.20	0.96
		(−4.85, 13.64)	(−5.42, 12.63)	(−2.60, 15.00)	(−5.58, 7.49)
	Black	**−15.41**	−2.71	−9.71	**6.86**
		(−27.14, -3.68)	(−12.77, 7.36)	(−19.89, 0.47)	(0.33, 13.38)
	Other	−1.37	−0.59	−2.04	0.32
		(−8.17, 5.44)	(−7.07, 5.88)	(−9.84, 5.76)	(−5.51, 6.15)
	South Asian	−3.92	−2.22	1.73	0.26
		(−13.18, 5.35)	(−12.51, 8.06)	(−7.82, 11.29)	(−6.43, 6.95)
Comorbidity Count		**−3.55**	**−3.43**	**−2.63**	**−2.32**
		(−4.63, -2.48)	(−4.68, -2.18)	(−3.70, -1.55)	(−3.12, -1.53)
Body Mass Index (ref: Normal)	Overweight	0.27	3.18	−2.89	1.06
		(−4.02, 4.55)	(−1.38, 7.73)	(−7.38, 1.59)	(−2.45, 4.56)
	Obese	−1.71	2.97	−2.82	1.73
		(−6.70, 3.28)	(−2.40, 8.33)	(−8.12, 2.48)	(−2.01, 5.48)

*all health domain scores were simultaneously assessed as dependent variables, with allowance for correlations between health domain scores;

**bolded estimates represent statistical significance (i.e. p < 0.05).

**Table 6:** Cohort-stratified path regression results; outcome: general health score*

		**Foot/Ankle**	**Elbow/Shoulder**	**Neck/Back**	**Hip/Knee**
		**(n = 349)**	**(n = 378)**	**(n = 454)**	**(n = 767)**
			Outcome: General Health Score		
Predictors			Beta Coefficients**		
			(95% Confidence Limits)		
Age		0.06	0.02	0.09	0.02
		(−0.09, 0.21)	(−0.12, 0.15)	(−0.04, 0.22)	(−0.08, 0.12)
Sex (ref: Male)	Female	1.81	−3.47	3.28	−0.81
		(−2.65, 6.26)	(−7.39, 0.45)	(−0.77, 7.34)	(−3.76, 2.14)
Income (ref: high)	Low	−4.97	−4.01	−5.11	**−5.18**
		(−11.42, 1.49)	(−9.72, 1.70)	(−11.80, 1.57)	(−9.72, -0.64)
	Missing	**−9.28**	−3.19	−2.72	−0.06
		(−16.18, -2.39)	(−9.38, 3.00)	(−6.98, 1.53)	(−4.22, 4.09)
Education (ref: >high school)	≤ high school	−4.66	−3.17	−2.51	−2.10
		(−9.92, 0.61)	(−7.18, 0.85)	(−6.59, 1.57)	(−5.34, 1.14)
Race (ref: White)	Asian	−0.07	−0.73	−4.07	**−8.45**
		(−10.79, 10.65)	(−9.34, 7.89)	(−12.89, 4.76)	(−14.93, -1.98)
	Black	**−13.75**	−6.64	−8.87	−1.07
		(−27.36, -0.14)	(−16.26, 2.97)	(−18.88, 1.15)	(−7.55, 5.40)
	Other	−3.47	−0.78	−7.33	2.89
		(−11.28, 4.34)	(−6.95, 5.40)	(−15.14, 0.48)	(−2.90, 8.67)
	South Asian	−0.96	**−10.35**	**−11.42**	0.21
		(−11.71, 9.79)	(−20.16, -0.54)	(−20.98, -1.85)	(−6.37, 6.78)
Comorbidity Count		**−3.27**	**−5.28**	**−4.35**	**−3.29**
		(−4.51, -2.04)	(−6.47, -4.09)	(−5.41, -3.28)	(−4.08, -2.51)
Body Mass Index (ref: Normal)	Overweight	−1.92	−1.30	−0.68	0.62
		(−6.86, 3.02)	(−5.64, 3.04)	(−5.17, 3.80)	(−2.85, 4.10)
	Obese	**−12.95**	−3.47	**−5.82**	−2.96
		(−18.71, -7.20)	(−8.56, 1.61)	(−11.12, -0.52)	(−6.67, 0.75)

*all health domain scores were simultaneously assessed as dependent variables, with allowance for correlations between health domain scores;

**bolded estimates represent statistical significance (i.e. p < 0.05).

For bodily pain (Table 3): Increasing age was generally associated with worse scores. While female sex was associated with worse scores, generally, the effects were much larger within elbow/shoulder and hip/knee group, where scores were 4–5 points lower for women, as compared to the foot/ankle group where the difference in scores between men and women was less than 0.5 points. Low income was associated with worse scores, and this effect was most notable for the foot/ankle group, where scores were 11 points lower for the low income group as compared to those with high income. Compared to Whites, Asians tended towards better scores across the cohorts. However, this was much more so among the elbow/shoulder group, where Asians had average scores 16 points higher than Whites. Being overweight or obese was generally associated with worse bodily pain scores. The negative effects of being obese were most pronounced for the foot/ankle group, with average scores 13 points lower for obese vs. normal BMI, compared to 4–7 points lower among the other groups.

For physical functioning (Table 4): Women had lower scores in three of the cohorts, (approximately 6–7 points lower for elbow/shoulder and hip/knee, and 4 points lower for neck/back), but this relationship was not seen in the foot/ankle cohort, in which mean scores were approximately 5 points higher among women. The negative effects of low income on physical functioning scores also varied across groups, with scores on average 10–14 points lower for the foot/ankle and elbow/shoulder group, compared to 1–5 points lower for the neck/back and hip/knee groups. Though not statistically significant, individuals of South Asian ethnicity generally had worse physical functioning scores compared to Whites. Again, obesity was associated with worse scores across the groups. However, these negative effects varied from 5 points lower among the elbow/shoulder group, to more than 14 points lower among the foot/ankle group, as compared to normal BMI.

For mental health (Table 5): Increasing age was associated with better mental health scores across the cohorts, while low income status was associated with worse scores. Compared to Whites, Asians trended towards better mental health scores. Blacks, however, had worse scores across three of the groups (15 points lower among the foot/ankle cohort), and better scores, nearly 7 points higher on average, within the hip/knee group. Significant effects for overweight and obese status were not found across the groups for mental health scores.

Finally, for general health (Table 6): Low income status and lower educational attainment were generally associated with worse scores across the groups. Compared to Whites, Asians trended towards worse scores within the hip/knee and neck/back cohorts, and Blacks towards worse scores within the three groups not including hip/knee, averaging nearly 14 points lower among foot/ankle compared to 6–8 points lower within elbow/shoulder and neck/back groups. South Asians scored on average 10–11 points lower within the elbow/shoulder and neck/back group, while showing no effects within foot/ankle and hip/knee groups. As was the case for each of the other health domain outcomes, higher comorbidity counts were associated with worse scores across all groups. Finally, obesity was associated with worse scores, but more so among the foot/ankle group, where scores were on average 13 points lower, compared to 3–6 points lower among the other cohorts.

## Discussion

We identified unique associations between patient characteristics and health status measures among patients dependent on the anatomical region for which musculoskeletal orthopaedic care was sought. Given that previous research has variably linked pre-surgical patient health status to patient-reported medical and surgical outcomes, compliance, willingness to consider surgery, and patient satisfaction [2-14,21,22], this knowledge can inform musculoskeletal health professionals’ efforts to provide patient- and cohort-targeted counsel that is more likely to have maximal positive effect on a range of outcomes. It also may contribute to the future development of targeted decision-aids.

Across the cohorts, average health domain scores were significantly lower (i.e. worse) than comparable estimates for the general Canadian population [23]. While distributional differences in demographic and socioeconomic characteristics and overweight and obese status were found across the cohorts, this heterogeneity did not account for the differences observed in health domain scores across the cohorts. Comparatively, individuals within the foot/ankle cohort generally had better scores, followed by the hip/knee and elbow/shoulder cohorts with worse, and relatively similar (except for physical functioning where hip/knees fared worse) scores. The neck/back cohort generally had worse scores across the health domains, with the exception of physical functioning, where estimates were equivalent to those for the hip/knee cohort.

Of particular significance in this study was the identification of differential ‘effect sizes’ for personal factors on health domain scores across the cohorts. For example, while the proportion of obese individuals was lowest among the foot/ankle cohort, the negative influence of being obese on health domain scores was generally greatest within this cohort. For example, for bodily pain and general health, the negative effect of obesity within the foot/ankle cohort was on average twice that found within the group with the next largest effect. This information should critically inform the clinician-patient educational interaction with respect to the relative importance attributed to different treatment modalities and self-management strategies.

Differential influences also were noted for level of income across the domains and cohorts. For example, low income status was associated with a 10–14 point lower score for physical functioning among the foot/ankle and elbow/shoulder cohort, compared to nearly no effect in neck/back and a 5-point lower score for hip/knee. For bodily pain, low income status was associated with an 11-point lower score for foot/ankle, compared to a 4–7 point lower score for the other groups. Such wide differentials across cohorts for the effects of low income on mental health and general health were not observed, however. Lower educational attainment was generally associated with poorer health domain scores, with relatively similar effects across the cohorts. Lower socioeconomic status and poorer health have been associated with greater unmet need for or decreased willingness to undergo elective orthopaedic surgery [24-27]. Additionally, low socioeconomic status can have potentially significant implications for access to services (e.g. physiotherapy) or supports or devices (e.g. orthotics), which the patient may have to pay for out-of-pocket [28,29]. Thus, these findings should inform the tailoring of the educational approach, recognizing that low SES differentially influences health status depending on the cohort.

Some important differences were observed in the influence of racial background on health domain scores across the cohorts. Asians trended towards better or similar health domain scores compared to Whites, except for worse scores for general health among the neck/back and hip/knee cohorts. While Blacks generally trended towards worse scores, notable differences were observed for mental health, where points were on average 15 points lower among foot/ankle, compared to 7 points better among hip/knee. For general health, points were on average 14 points lower for Blacks within foot/ankle, compared to 6–9 points lower for elbow/shoulder and neck/back, and nearly no effect within hip/knee. South Asian ethnicity, as compared to White, showed more difference across health domains than across cohorts. For instance, little effect was observed for bodily pain, whereas generally negative effects were observed for physical functioning scores. On the other hand, strong negative effects were found for general health in the elbow/shoulder and neck/back cohorts (average of 10.5 points lower), compared to no effect within foot/ankle and hip/knee. These differential findings, particularly with respect to mental and general health, across racial groups can have significant implications for the patient educational process and counsel. Generally, mental well-being, overall health status, and cultural perceptions have been shown to be associated with expectations of and decisions to undergo surgery, post-surgical patient-reported outcomes, compliance, and uptake or maintenance of self-management/self-care activities [7,12,24,26,30-40].

This study was undertaken to better characterize the patient seeking elective musculoskeletal orthopaedic care. The study sample was recruited and assessed prior to consultation, and consequently the specific degenerative diagnosis and disease stage was not considered. The advantage of this is that the results are broadly generalizable to the population seeking care. However, a limitation of not considering this is that differential distributions between cohorts may explain overall differences in health statuses between cohorts. The likely influences within the specific cohorts are unknown, however. Assuming greater homogeneity within specific cohorts than across cohorts would lead us to speculate that diagnostic consideration would not substantively alter our conclusions, particularly as the health outcome measures were purposively selected to be generic and non-disease specific. Finally, this study was limited to orthopaedic patients recruited from an academic, tertiary care centre, which may limit generalizability. However, the included degenerative diagnostic categories and patient mix included are typical of community practice and not specific to tertiary care.

## Conclusion

We report that there is substantial heterogeneity across musculoskeletal cohorts both in health status and in the association between personal characteristics and various health domains. Given known associations between patient health status and medical and surgical outcomes, this suggests that patient- and cohort-specific education is likely to achieve superior results on a range of patient outcomes.

## Competing interests

The authors declare that they have no competing interests.

## Authors’ contributions

All authors made substantial contributions to study conception and design. RG and YRR made substantial contributions to the acquisition of data. AVP, RG, and YRR contributed to the analysis and interpretation of data. AVP drafted the manuscript and all authors revised it critically for important intellectual content. All authors have given final approval of the version to be published.

## Authors’ information

Arthritis Program Members: Anthony V Perruccio, PhD, Rajiv Gandhi, MD, MSc, Y Raja Rampersaud, MD, J Roderick Davey, MD, Johnny TC Lau, MD MSc, Stephen Lewis, MD Msc, K Wayne Marshall, MD PhD, Darrel J Ogilvie-Harris, MB, Khalid Syed, MD, Christian Veillette, MD MSc, Nizar N Mahomed, MD DSc.

## Pre-publication history

The pre-publication history for this paper can be accessed here:

http://www.biomedcentral.com/1471-2474/14/83/prepub
